# Reduction in Noise-Induced Functional Loss of the Cochleae in Mice with Pre-Existing Cochlear Dysfunction Due to Genetic Interference of Prestin

**DOI:** 10.1371/journal.pone.0113990

**Published:** 2014-12-08

**Authors:** Qunfeng Cai, Bo Wang, Donald Coling, Jian Zuo, Jie Fang, Shiming Yang, Krystal Vera, Bo Hua Hu

**Affiliations:** 1 Center for Hearing and Deafness, University at Buffalo, Buffalo, New York, United States of America; 2 Department of Otolaryngology and Head & Neck Surgery, Institute of Otolaryngology, Chinese PLA General Hospital, Beijing, China; 3 Developmental Neurobiology, St. Jude Children's Research Hospital, Memphis, Tennessee, United States of America; Osaka University Graduate School of Medicine, Japan

## Abstract

Various cochlear pathologies, such as acoustic trauma, ototoxicity and age-related degeneration, cause hearing loss. These pre-existing hearing losses can alter cochlear responses to subsequent acoustic overstimulation. So far, the knowledge on the impacts of pre-existing hearing loss caused by genetic alteration of cochlear genes is limited. Prestin is the motor protein expressed exclusively in outer hair cells in the mammalian cochlea. This motor protein contributes to outer hair cell motility. At present, it is not clear how the interference of prestin function affects cochlear responses to acoustic overstimulation. To address this question, a genetic model of prestin dysfunction in mice was created by inserting an internal ribosome entry site (IRES)-CreER^T2^-FRT-Neo-FRT cassette into the prestin locus after the stop codon. Homozygous mice exhibit a threshold elevation of auditory brainstem responses with large individual variation. These mice also display a threshold elevation and a shift of the input/output function of the distortion product otoacoustic emission, suggesting a reduction in outer hair cell function. The disruption of prestin function reduces the threshold shifts caused by exposure to a loud noise at 120 dB (sound pressure level) for 1 h. This reduction is positively correlated with the level of pre-noise cochlear dysfunction and is accompanied by a reduced change in *Cdh1* expression, suggesting a reduction in molecular responses to the acoustic overstimulation. Together, these results suggest that prestin interference reduces cochlear stress responses to acoustic overstimulation.

## Introduction

Acoustic overstimulation is a common cause of sensory cell damage in the cochlea. While the magnitude of acoustic trauma is associated with the properties of noise, the final outcome of cochlear degeneration is also related to the functional status of the cochlea at the time of noise exposure [1]–[3]. Various pathological conditions, such as aging degeneration, ototoxicity and acoustic trauma, can compromise cochlear function, which in turn alters cochlear responses to subsequent acoustic overstimulation. For example, drugs that have ototoxic effects on the sensory cells can either potentiate acoustic trauma [4] or protect the cochlea from the trauma [5], [6]. During age-related degeneration, cochlear susceptibility to noise demonstrates an inter-species difference. Species showing an early onset of cochlear dysfunction appear to be more susceptible to acoustic trauma than those with a later onset of aging degeneration [7]. However, within a species, the older subjects appear to have a similar susceptibility to acoustic trauma compared with the young subjects [8], [9]. For subjects with a prior history of noise injury, cochlear responses to a subsequent noise exposure depend on the profile of the prior noise impacts. Conditioning exposure to a moderate level of noise toughens the ear against subsequent traumatic noise exposure [10], [11]. Traumatic noise, on the other hand, potentiates cochlear damage to subsequent noise injury [12]. This effect occurs at the frequency region that is not damaged by the initial noise trauma [1]. Together, these observations suggest that the pre-existing cochlear dysfunction can affect the pattern of subsequent cochlear degeneration due to acoustic overstimulation.

At present, knowledge on the impacts of genetic hearing losses on cochlear responses to acoustic injury is limited. Several studies have documented that alteration of cochlear genes potentiates noise-induced cochlear damage. Targeted deletion of the cytosolic Cu/Zn-superoxide dismutase gene (Sod1) and the cellular glutathione peroxidase gene (Gpx1) increases the susceptibility of the subjects to noise injury [13]. Deficiency of the plasma membrane calcium ATPase isoform 2 gene (PMCA2) and a sodium-dependent glutamate/aspartate transporter gene (GLAST) also increases the susceptibility of the cochlea to acoustic trauma [14], [15]. The fact that all these genes have functional roles in inner ear biology suggests that interference of functional genes of the cochlea potentiates noise-induced hearing loss. So far, it is not clear whether interference of outer hair cell (OHC) genes could have a similar impact on cochlear responses to acoustic injury.

Prestin is the motor protein of OHCs coded by the solute carrier anion transporter family 26, member 5 gene (SLC26A5)[16]. This gene is expressed along the basolateral membrane of OHCs and contributes OHC motility. Prestin knockout compromises hearing sensitivity by 45–60 dB [17], [18] and causes the loss of the voltage-dependent stiffness and piezoelectrical property of OHCs [19], suggesting a functional role for prestin in maintenance of cochlear function. At present, it is not known how cochlear dysfunction due to prestin interference alters cochlear responses to acoustic trauma.

In the current study, we used a mouse model of prestin interference created by the insertion of an internal ribosome entry site (IRES)-CreER^T2^-FRT-Neo-FRT cassette into the prestin locus after the stop codon. The homozygous mice exhibit diverse levels of hearing dysfunction, offering us an opportunity to generate a gradient disruption of cochlear function and to investigate this disruption's impact on cochlear responses to acoustic overstimulation. The study demonstrated that the interference of prestin function led to reduction in hearing sensitivity with large individual variation. Unlike many other causes of pre-existing hearing loss, the prestin-associated hearing loss led to reduction in noise-induced threshold shifts. The level of this reduction in threshold shifts was correlated with the level of the prestin-disruption-induced cochlear dysfunction. Moreover, the level of noise-induced molecular responses of the cochleae was reduced. Together, these observations suggest that interrupting prestin function reduces cochlear stress responses to acoustic overstimulation.

## Methods

### Subjects

Prestin-CreER^T2^ knockin mice (4–8 weeks old, male and female) were used to assess the effect of OHC dysfunction on cochlear responses to acoustic trauma. The breeder mice were provided by Dr. Jian Zuo, St. Jude Children's Research Hospital. The colony was established at the Lab Animal Facility in the University at Buffalo. The wild-type control mice were C57BL/6J mice (4–8 weeks old, male and female, the Jackson Laboratory, Bar Harbor, ME). The number of animals used for each experimental condition will be described in the Results section. Procedures involving the use and care of the animals were approved by the Institutional Animal Care and Use Committee of the State University of New York at Buffalo.

### The genotyping of Prestin-CreER^T2^ knockin mice

The genotypes of the Prestin-CreER^T2^ knockin mice were confirmed using a genotyping method that has been described before [20]. Briefly, a piece of tail was collected and lysed using a lysis reagent (DirectPCR Lysis, Viagen Biotech Inc, Los Angeles, CA, USA) containing freshly prepared proteinase K in a hybridization oven at 55°C for 6 h. Then, the crude lysate was incubated at 85°C for 45 min in a water bath. The lysate was transferred to a PCR tube, and gDNAs were amplified using PCR. The PCR products were separated by gel electrophoresis. The primers used were the following: 5′-CACAAGTTGTGAATGACCTC-3′, 5′-GTTAAAGAGCGTAATCTGGAACA-3′ and 5′-TAACTGCTAGCATTTCCCTT-3′.

### Auditory brainstem responses

Auditory brain response (ABR) measurements were performed before and at multiple time points after the noise exposure (see the Results for the details on the time points of the measurement) using a procedure that has been described before [21]. Briefly, the animals were anesthetized with an intraperitoneal injection of a mixture of ketamine (87 mg/kg) and xylazine (3 mg/kg). The body temperature was maintained at 37.5°C with a warming blanket. Stainless-steel needle electrodes were placed subdermally over the vertex (non-inverting input) and posterior to the stimulated and non-stimulated ears (inverting input and ground) of the animal. The ABRs were elicited and recorded using an evoked potential measurement system (TDT, Tucker-Davis Technologies, Alachua, FL, USA). The responses were elicited with tone bursts at 5, 10, 30 and 40 kHz (0.5 ms rise/fall Blackman ramp, 1 ms duration, alternating phase) at the rate of 21/s. The responses were filtered (100–3000 Hz), amplified and averaged using TDT hardware and software. The ABR threshold was defined as the lowest intensity that reliably elicited a detectable response. To define the changes in auditory function, threshold shifts were calculated using the pre-treatment thresholds as the baseline. The ABR measurements were performed by a single observer and the observer was not blinded to the experimental conditions.

In certain animals, the ABR thresholds became undetectable at the 2-h time point after the noise exposure, even using the maximal output of our ABR testing system (105 dB SPL). To evaluate the auditory function at this circumstance, we used two complementary methods. First, we counted the frequency points that the ABR thresholds were detectable for each animal, and then we calculated the percentage of the detectable frequency points among the four tested frequencies (5, 10, 30 and 40 kHz) for each animal. The percentages were averaged for each group, and the group data were compared using one-way analysis of variance (ANOVA). Second, we rendered the threshold value 105 dB (the maximal output of our ABR testing system) for each undetectable frequency point, and we calculated the average threshold for each group. The group data were compared using one-way ANOVA. This strategy could certainly have created an analysis bias (underestimation of the group difference). However, such a bias did not affect the conclusions of the analysis (see the Results section, “Homozygous mice exhibit less threshold shifts after exposure to the intense noise”, for detailed explanations).

### Distortion product otoacoustic emission measurement

Distortion product otoacoustic emission (DPOAE) was measured to assess OHC function using a procedure that was described in our previous publication [22]. DPOAE was measured using a TDT System. DPOAEs were elicited by two primary tones, F_1_ and F_2_, with an F_2_/F_1_ ratio of 1.2. L_1_ was set 10 dB higher than L_2._ F_2_ frequencies were set at 8 and 16 kHz for the mice. DPOAE input/output functions were obtained by decreasing the L_2_ intensity in 5-dB steps. Thirty-two sweeps were presented at each test level. These responses were then stored and displayed on a computer. The input/output functions of DPOAE were averaged for each tested frequency. The threshold of DPOAE was defined as 3 dB above the noise floor.

### Acoustic overstimulation

A continuous noise (1–7 kHz) at a 120 dB sound pressure level (SPL, re 20 µPa) for 1 h was used to stimulate the cochleae. The noise signal was generated using a real-time signal processor (RP2.1, Tucker Davis Technologies, TDT). The signal was routed through an attenuator (PA5 TDT, Alachua, FL, USA) and a power amplifier (Crown XLS 202, Harman International Company) to a loudspeaker (NSD2005-8, Eminence) positioned 30 cm above the animal's head. The noise level at the position of the animal's head in the sound field was calibrated using a sound level meter (Larson Davis, APCB Piezotronics Div., LD-PCB, model 800 B), a preamplifier (LD-PCB, model 825), and a condenser microphone (Larson and Davis, LDL 2559). The animals were individually exposed to the noise in a holding cage. This noise paradigm was employed in our previous investigation and is able to generate functional and morphological changes in the cochlea [23], [24].

### Cochlear tissue collection

Upon the completion of the final functional evaluation, the animals were decapitated under deep anesthesia with CO_2_. The cochleae were quickly removed from the skull and processed for subsequent analyses.

#### For transcriptional analysis

A sensory cell-enriched tissue sample was collected using a method described in our recent publication [25]. Briefly, the cochlea was placed in an ice cold Dulbecco's phosphate buffer saline solution (DPBS, GIBCO) as an initial preparation to open the cochlea and to remove modiolus and lateral wall tissues. Then, the cochlea was transferred into a PCR tube that contained 0.6 ml of an RNA-stabilizing reagent (RNAlater, Qiagen, Valencia, CA) and was stored at 4°C overnight. Then, microdissection was performed in the RNAlater solution to collect the organ of Corti tissue from the apical portion of the first cochlear turn. The collected tissue contained sensory cells (inner hair cells and OHCs), pillar cells (both inner and outer), Deiters cells, inner phalangeal cells and inner border cells.

#### For immunolabeling and pathological assessment

The cochleae were collected and perfused through the round window with 10% buffered formalin (Fisher Scientific). The cochleae were immersed in the fixative overnight and then transferred to a dish containing 10 mM PBS for dissection. Cochlear dissection was performed to collect the cochlear sensory epithelium for surface preparation.

### qRT-PCR analysis

Total RNA was extracted from the collected tissues using the RNeasy Micro Kit (Qiagen) as previously described [21]. The transcriptional expression levels of prestin *(Slc26a5*) and Cdh1 were examined using pre-developed TaqMan gene expression primer/probe assays (Applied Biosystems). The prestin mRNA was examined in Prestin-CreER^T2^ mice (+/+) and wild-type mice to determine whether Prestin-CreER^T2^ knockin could affect the transcriptional expression of prestin. *Cdh1* expression was examined to assess the molecular stress responses of the organ of Corti to acoustic overstimulation in the Prestin-CreER^T2^ mice (+/+). The isolated total RNAs from 10 cochleae (for the prestin analysis) and 9 cochleae (for the *Cdh1* analysis) of Prestin-CreER^T2^ homozygous mice were reverse transcribed using a High Capacity cDNA reverse transcription kit (Applied Biosystems). qRT-PCR was performed on a MyIQ-two color real time PCR detection system (BioRad, Hercules, CA). Pre-developed *Actin, GAPDH and Hprt1* gene expression assays (Applied Biosystems) were used as endogenous controls.

### Immunohistology of prestin

Immunohistochemistry was used to examine the immunoreactivity of prestin in the OHCs of the Prestin-CreER^T2^ homozygous mice (n = 4 cochleae) and wild-type mice (n = 5 cochleae). The cochleae were fixed with 10% buffered Formalin for 2 h. After dissection in 10 mM PBS, the sensory epithelium tissues were collected. The tissues were then permeabilized with 0.2% Triton X-100 in PBS for 30 min, blocked with 10% donkey serum in PBS and then incubated with goat primary antibody against prestin (1∶200, sc-22692, Santa Cruz Biotechnology, Inc.) at 4°C overnight. The tissues were rinsed with PBS and incubated with a secondary antibody (Alexa Fluor 488 donkey anti-goat, 1∶500, Life Technologies) for 1 h. Confocal microscopy was performed to examine the expression pattern of prestin in the OHCs.

### Assessment of sensory cell damage

The numbers of missing sensory cells in the organs of Corti were quantified using f-actin staining in the cuticular plates [26]. The absence of fluorescence in the cuticular plate region indicates a loss of cells. Specifically, the formalin-fixed cochleae collected from the Prestin-CreER^T2^ mice and wild-type mice were dissected in 10 mM PBS, and the sensory epithelium tissues were collected. The tissues were permeabilized with 0.2% Triton X-100 in PBS for 30 min, blocked with 10% goat serum in PBS and incubated with Alexa 488-labeled phalloidin (Invitrogen) for 30 min at room temperature. The number of missing outer hair cells was quantified by a single observer and this observer was not blinded to the experimental conditions.

### Statistical analyses

All values are represented as the mean value and ± standard deviation. The significance of changes or differences between conditions was examined using two statistical programs, SigmaStat for Windows V. 3.5 or Prism 5 for Windows. Based on the experimental designs, one-way ANOVA, two-way ANOVA, unpaired Student's *t* test, parried Student's *t* test and Pearson's correlation analysis (see the Results section for details) were used in the assessment. The identification of significant ANOVA effects and interactions was followed by appropriate pairwise comparisons, using Tukey or Bonferroni. Results were considered statistically significant only if the P value was <0.05.

## Results

### Prestin-CreER^T2^ homozygous mice exhibit a reduced hearing sensitivity

**Figure 1:**
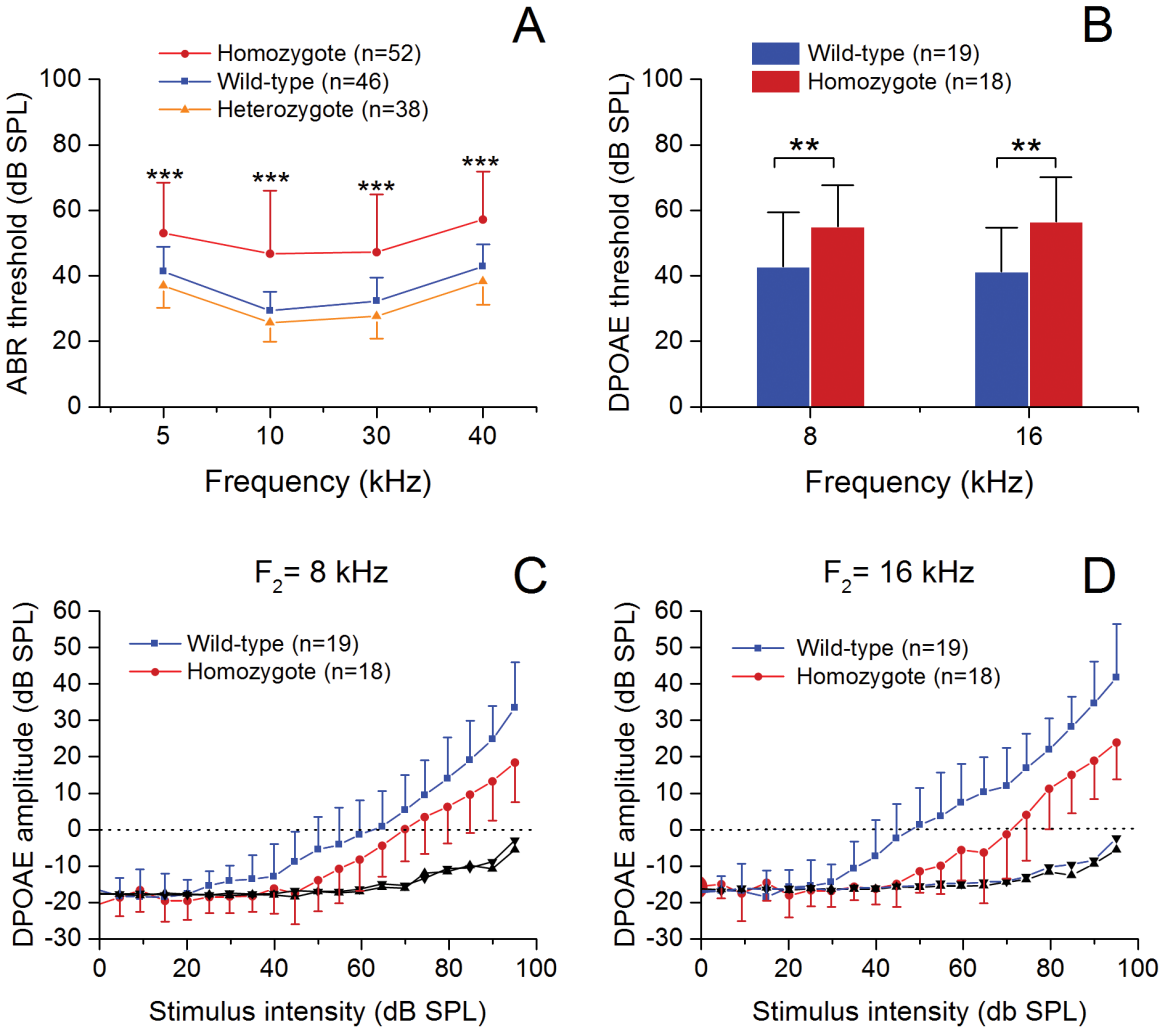
Prestin-CreER^T2^ mice (+/+) exhibit a reduced hearing sensitivity. A Comparison of the ABR thresholds of Prestin-CreER^T2^ homozygotes (+/+), heterozygotes (+/-) and wild-type mice at the four tested frequencies. The homozygous mice exhibit elevated thresholds compared with the wild-type mice (two-way ANOVA, *F_1,384_* = 121.0, *P*<0.001; *** indicates *P*<0.001 tested by Tukey test). B Comparison of the thresholds of DPOAEs between the Prestin-CreER^T2^ homozygous mice and wild-type mice for the two tested frequencies (F_2_ = 8 and 16 kHz). The homozygous mice show elevated thresholds compared with the wild-type mice (Two-way ANOVA, *F_1,70_* = 17.2, *P*<0.001; ** indicates *P*<0.01 tested by Tukey test). C and D The comparisons of the input/output functions of the DPOAE between the Prestin-CreER^T2^ homozygous mice and wild-type mice at the two tested frequencies: F_2_ = 8 kHz and F_2_ = 16 kHz. The homozygous mice display the reduced amplitudes of DPOAE at both tested frequencies compared with those observed in the wild-type mice. These results suggest that Prestin-CreER^T2^ knockin affects cochlear function by interfering with OHC function. n: the number of the cochleae used for each group.

We measured the threshold of ABR responses to determine the baseline hearing sensitivity of the knockin mice and the wild-type mice. The differences in the thresholds between the heterozygous and wild-type mice were less than 5 dB for the four tested frequencies (Fig. 1A). This level of difference is considered biologically insignificant. In contrast, the homozygous mice exhibited a flat threshold elevation with an average of 16–21 dB shift across the four tested frequencies, compared with those of the wild-type mice (Fig. 1A; two-way ANOVA, *F_1,384_* = 121.0, *P*<0.001; Tukey test, *P*<0.001 for all of the test frequency points). These observations suggest that insertion of CreER^T2^ into one allele does not induce a significant loss of hearing sensitivity, whereas the insertion of Prestin-CreER^T2^ into two alleles reduces the hearing sensitivity. These results are consistent with the results of a previous observation that prestin knockout mice (-/-) showed significant hearing loss, whereas the heterozygous subjects (+/-) exhibited only a few decibels of hearing loss [17], [27]. They are also consistent with the results that one hypomorphic allele of prestin knockin does not affect overall hearing sensitivity of the subjects [28].

DPOAE is an acoustic signal generated by OHCs. Its amplitude reflects the OHC function. To determine whether homozygous mice have reduced OHC function, we measured the thresholds and the input/output function of the DPOAEs. The DPOAE threshold was defined as the L_2_ level at which the amplitude of the responses was 3 dB above the noise floor. Compared with those in the wild-type mice, the average thresholds in the homozygous mice were elevated by 12 dB for 8 kHz and 15 dB for 16 kHz (Two-way ANOVA, *F_1,70_* = 17.2, *P*<0.001; Tukey test, *P* = 0.009 for 8 kHz and *P = *0.002 for 16 kHz; Fig. 1B). Moreover, the average input/output function of the DPOAEs in the homozygous mice shifted in a parallel fashion compared with those of the wild-type mice (Figs. 1C and 1D), suggesting a reduction in OHC function. Collectively, these observations suggest that Prestin-CreER^T2^ knockin affects cochlear function by interfering with OHC function.

### The Prestin-CreER^T2^ knockin homozygous mice exhibit large inter- and intra-subject variation in cochlear dysfunction

**Figure 2:**
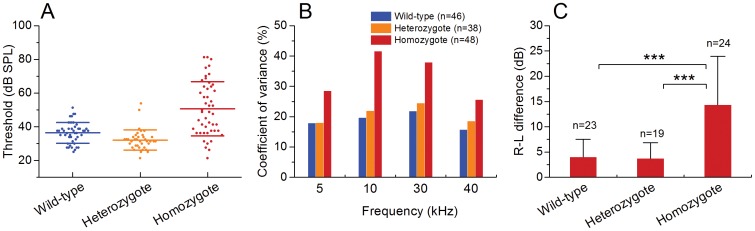
Prestin-CreER^T2^ knockin homozygous mice exhibit large inter- and intra-subject variation in ABR thresholds. **A** A scatter plot shows the distribution of the ABR thresholds of individual ears in the Prestin-CreER^T2^ mice (+/+ and +/-) and the wild-type mice. One dot represents the average threshold of one ear calculated from the four tested frequencies. Notice that the ABR thresholds of the homozygous mice are more dispersedly distributed, from 21 to 81 dB SPL. By contrast, the thresholds of the heterozygous mice and the wild-type mice are much less diverse (25–51 dB for the wild-type mice and 21–54 dB for the heterozygous mice). The three lines in each group indicate the mean and ± standard deviation. **B** Comparison of the values of coefficient of variation among the three groups of mice. Homozygous mice display relatively larger values compared with those of the heterozygous and wild-type mice for all four tested frequencies. **C** Comparison of the levels of the two ear differences (the right ear vs. the left ear of the same animal) in the average of ABR thresholds of the four tested frequencies among the three groups of mice. The threshold differences between the two ears in the homozygous mice is significantly greater than those observed for the heterozygous mice or the wild-type mice (One-way ANOVA, *F_2,63_ = *20.52, *P*<0.001; *** indicates *P*<0.001 tested by Tukey test). n: the number of the animals used for each group.

The ABR thresholds varied across individual subjects in all three groups of subjects. However, the variation in the homozygous mice was significantly greater than those in the wild-type and heterozygous mice (Fig. 2A). To quantify the variations, we calculated the coefficient of variation for each group and found that the homozygous mice exhibited larger values than those observed for the heterozygous and wild-type mice in the four tested frequencies (Fig. 2B).

We further examined the magnitudes of the interaural differences in the thresholds of ABRs to determine whether the individual variation occurs between the two ears of the individual animals. As shown in Fig. 2C, the interaural differences are significantly greater in the homozygous mice than those in the heterozygous and wild-type mice (one-way ANOVA, *F_2,63_ = *20.52, *P*<0.001, Tukey test, *P*<0.001). These results suggest that the homozygous mice display not only large inter-animal variation but also large intra-animal variation.

The dynamic range between the highest and the lowest ABR thresholds was 60 dB for the homozygous mice. Because of the concern that using the highest and lowest values for the range assessment may lead to an analysis bias due to the presence of extreme values, we averaged the top 10% lowest (n = 5 cochleae) and highest (n = 5 cochleae) ABR thresholds. The averaged threshold from the five cochleae with the lowest thresholds was 27.5±3.9 dB. This value was lower than, but within one standard deviation of, the average of the thresholds for heterozygous mice (32.1±6.1 dB), suggesting that these ears had a similar hearing sensitivity as those of the heterozygous mice. The average of the top 10% highest thresholds was 79.3±2.0 dB. The range between the top 10% lowest and the top 10% highest thresholds was 51.8 dB. This level of difference is comparable to the level of hearing loss (45-65 dB) caused by prestin knockout [17], [18], [27], suggesting that the maximal hearing impact of Prestin-CreER^T2^ knockin is equivalent to the level of hearing impact induced by prestin knockout. Together, these results suggest that Prestin-CreER^T2^ knockin can cause diverse levels of hearing impairment. This large individual variation provided us with an opportunity to generate an animal model of gradient OHC dysfunction.

### Prestin expression remains consistent in the Prestin-CreER^T2^ knockin homozygous mice that have diverse levels of hearing sensitivity

**Figure 3:**
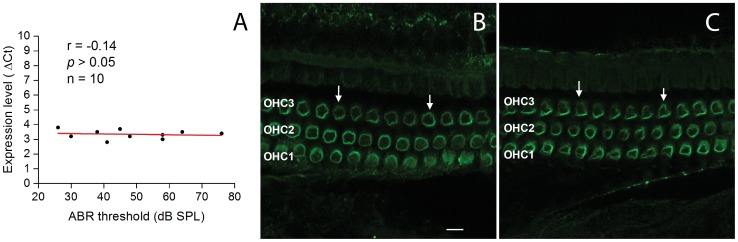
Prestin expression remains consistent in the Prestin-CreER^T2^ knockin homozygous mice showing diverse levels of hearing sensitivity. **A** Correlation between the transcriptional expression levels of prestin and the ABR thresholds of the same ears in the Prestin-CreER^T2^ knockin homozygous mice. The total RNAs were extracted from the organ of Corti tissues, and the prestin mRNA expression level was normalized to the reference gene (*Hprt1*). There is no significant correlation between the level of prestin expression and the level of ABR threshold (Pearson's correlation analysis, *r* = −0.140, *P* = 0.699), indicating that the prestin expression level remains consistent among the ears with diverse threshold levels of ABRs. **B** A typical image of prestin immunoreactivity in the cochlear sensory epithelium of a wild-type mouse. The circular immunolabeling in the three rows of OHCs, indicated by the arrows, is clearly visible. OHC1, OHC2 and OHC3 indicate the first, second and third rows of OHCs, respectively. Bar  = 20 µm. **C** A typical image of prestin immunoreactivity in the cochlear sensory epithelium of a Prestin-CreER^T2^ knockin homozygous mouse that had a high level of the average ABR threshold (81 dB SPL). The pattern of prestin immunoreactivity is similar to that observed in the control ear (see Fig. 3B). Note that the fluorescence intensity of the prestin immunolabeling appears not be homogenous across certain OHCs in both (**A**) and (**B**), which is due to the off-focus of the confocal images.

To determine whether homozygous mice that had diverse levels of hearing dysfunction exhibit corresponding variation in their prestin expression levels, we examined both the abundance of prestin mRNAs using qRT-PCR and the expression pattern of the prestin protein using immunohistochemistry. For the mRNA analysis, we collected sensory cell-enriched tissues containing only the sensory cells and their neighboring supporting cells to improve the spatial specificity of this cell-specific analysis [25]. The average ΔCt value of the prestin mRNAs (re: the expression level of a reference gene, *Hprt1*) was 3.3 in the homozygous mice (n = 10) and 5.0 in the wild-type mice (n = 3); this difference was statistically significant (Student's *t* test, t = 7.84, P<0.001), suggesting a reduction in the expression level of prestin in the homozygous mice. However, the variation in the ΔCt values of the prestin mRNAs in homozygous mice was small (standard deviation  = 0.3). The Pearson's correlation analysis showed that this small variation is not correlated with the ABR thresholds of the ears (*r* = −0.140, *P* = 0.699, Fig. 3A), suggesting that the level of hearing loss is not related to the expression level of prestin mRNA in the homozygous mice.

Immunohistochemistry for prestin was performed using a surface preparation of the cochlear sensory epithelium. Using confocal microscopy, we examined the prestin immunoreactivity in the OHCs of the first cochlear turn, the major site of acoustic injury. We found a typical circular distribution of prestin immunoreactivity in the OHCs of wild-type mice (Fig. 3B). The Prestin-CreER^T2^ knockin homozygous mice displayed similar prestin immunoreactivity (Fig. 3C). We found no detectable difference in prestin immunoreactivity between the cochleae with and without threshold shifts (data not shown). These observations suggest that prestin-CreER^T2^-knockin-induced hearing loss is not mediated by the expression interference of the gene, although we did not quantitatively measure prestin protein levels.

### Homozygous mice exhibit fewer threshold shifts after exposure to the intense noise

To investigate the effects of OHC dysfunction on cochlear responses to acoustic overstimulation, we exposed the animals to a 120 dB (SPL) broadband (1-7 kHz) noise for 1 h and examined the changes in the ABR thresholds at two time points (2 h and 7 d) after the noise exposure. The 2-h time point represents the acute cochlear stress response, and the 7-d time point represents the chronic/recovery phase of cochlear pathogenesis.

#### Acute changes in hearing threshold at 2-h post-noise exposure

**Figure 4:**
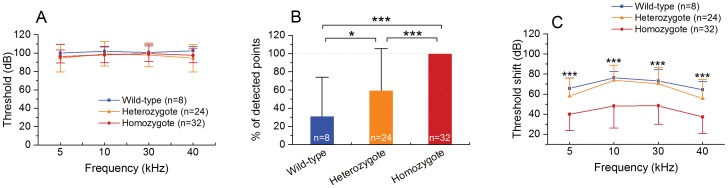
The Prestin-CreER^T2^ knockin homozygous mice exhibit fewer threshold shifts in the acute phase of cochlear pathogenesis (2 h after the noise exposure). **A** Elevation of ABR thresholds at 2 h after the noise exposure. **B** Comparison of the percentages of the average detectable ABRs among the three groups (the Prestin-CreER^T2^ homozygous, heterozygous and wild-type groups). Detectable ABRs were defined as ABRs that could be visually identified within the intensity range of the stimuli. The percentage of detection is 100% for the homozygous mice. By contrast, the values are 40.6±45.9% for the wild-type mice and 60.8±42.8% for the heterozygous mice. Both are significantly lower than that of homozygous mice (one-way ANOVA; *F_2,65_* = 23.039, *P*<0.001; *** indicates *P*<0.001 tested by Tukey test). In addition, the heterozygous mice had a greater detectable rate than that observed for the wild-type mice (* indicates *P* = 0.045 tested by Tukey test). **C** Comparison of the ABR threshold shifts among the Prestin-CreER^T2^ mice (+/+ and +/-) and wild-type mice. The levels of the threshold shift of the homozygous mice are significantly smaller than those of the heterozygous and wild-type mice (two-way ANOVA, two-way ANOVA, *F_2, 244_* = 56.11, *P*<0.001; *** indicates *P*<0.001 tested by Tukey test). These results indicate that the Prestin-CreER^T2^ knockin homozygous mice have a reduced level of hearing loss at the early phase of acoustic trauma.

Exposure to the loud noise caused significant shifts in the ABR thresholds across all tested frequencies. The occurrence of such broad damage was associated with the characteristics of the noise frequency and intensity used in the current study. In certain tested frequencies, the ABR thresholds became undetectable, even using the maximal intensity of acoustic signals available for our ABR testing system (105 dB SPL). Consequently, the thresholds reached to a ceiling level (Fig. 4A). We therefore counted the frequency points at which ABRs were present and calculated the percentage of the detectable points over the total number of tested points for each ear. The average percentages of the detectable points for the heterozygous and wild-type groups were 40.6±45.9% and 60.8±42.8%, respectively. By contrast, the percentage was 100% for the homozygous mice. The differences between the homozygous and heterozygous/wild-type mice were statistically significant (Fig. 4B; one-way ANOVA, *F_2,65_* = 23.039, *P*<0.001; Tukey test, *P*<0.001), indicating that the heterozygous and wild-type mice had greater threshold shifts after the acoustic trauma. There was also a difference between the wild-type and heterozygous mice (Fig. 4B; Tukey test, *P* = 0.045). We compared the threshold shifts among the three groups and found that the homozygous mice displayed lower threshold shifts compared with the wild-type and heterozygous mice (Fig. 4C, two-way ANOVA, *F_2,244_* = 56.11, *P*<0.001; Tukey test, *P* = 0.0007 to 0.0001). It should be noted that, for this analysis, all undetectable testing points were given a value of 105 dB, so that the statistical analysis could be performed. While this strategy of data analysis can lead to underestimations of the difference between the homozygous and heterozygous/wild-type mice, this underestimation does not affect our conclusion that homozygous mice had less hearing loss than heterozygous or wild-type mice in the acute phase of cochlear dysfunction.

**Figure 5:**
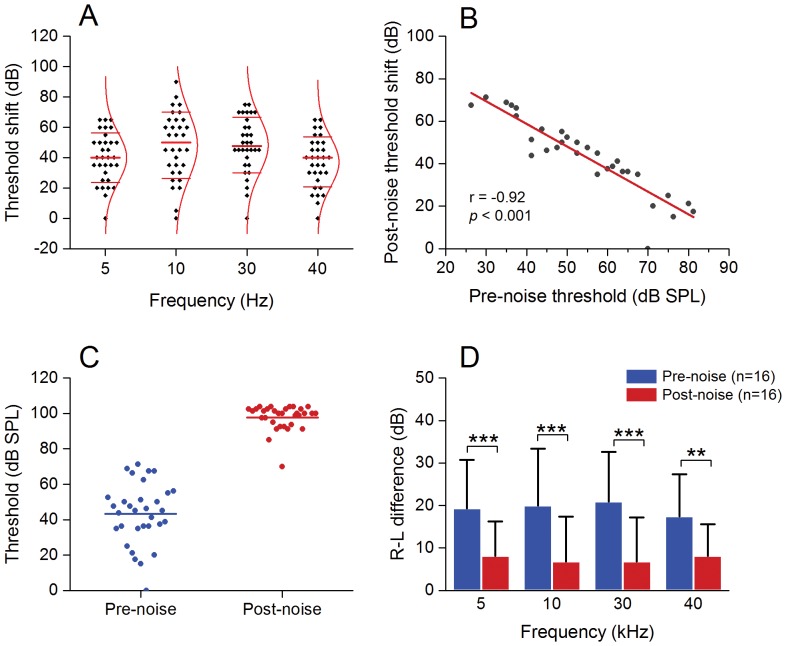
The level of noise-induced ABR threshold shifts is correlated with the level of pre-noise cochlear dysfunction in Prestin-CreER^T2^ homozygous mice. **A** A scatter plot shows significant individual variation in the levels of the threshold shifts of ABRs examined at 2 h post-noise exposure in the ears of Prestin-CreER^T2^ homozygous mice. The three horizontal lines in each frequency indicate the mean and ± standard deviation. The curve line represents the distribution curve of the data. One dot represents the averaged threshold of one ear from the thresholds of the four tested frequencies (5, 10, 30 and 40 kHz). **B** Correlation between the average pre-noise thresholds and post-noise threshold shifts of ABRs in the Prestin-CreER^T2^ homozygous mice. There is a negative correlation between the two variables (Pearson's correlation analysis, *r* = −0.92, *p*<0.001), indicating that, with the increase in the level of pre-noise hearing dysfunction, the noise-induced threshold shift decreases. **C** Comparison of the magnitude of individual variation in ABR thresholds, measured before and after the acoustic overstimulation in the Prestin-CreER^T2^ homozygous mice. One dot represents the threshold of one ear, as averaged from the thresholds of the four tested frequencies. The horizontal lines represent the means of the average ABR thresholds. Notice that the level of individual variation became smaller after the noise exposure. **D** Comparison of the levels of the two-ear difference in the ABR thresholds examined before and after the acoustic overstimulation. The levels of difference are significantly reduced after the acoustic trauma (two-way ANOVA, *F_1, 120_* = 41.47, *P*<0.001; *** indicates *P*<0.001 and ** indicates *P*<0.01 tested by Tukey test). n: the number of ears used for each condition.

The levels of the noise-induced threshold shift varied significantly across individual ears in the homozygous mice (Fig. 5A). To determine whether the levels of threshold shifts were correlated with the pre-noise hearing thresholds, we conducted a Pearson's correlation analysis. This analysis revealed a negative correlation between the pre-noise thresholds and post-noise threshold shifts (Fig. 5B, *r* = −0.92, *P*<0.001), suggesting that ears with a greater pre-noise hearing loss had less of a post-noise threshold shift. Together, these analyses indicate that the pre-noise elevation of the ABR thresholds attenuated the threshold shifts after the noise exposure.

While the individual cochleae had large threshold variations before the noise exposure, the level of variation became smaller after the noise exposure (Fig. 5C). The interaural difference was also reduced after the noise exposure (Fig. 5D, two-way ANOVA, *F_1,120_* = 41.47, *P*<0.001; Tukey test, *P* = 0.0031 for 5 kHz; *P* = 0.0007 for 10 kHz, *P* = 0.0003 for 30 kHz and *P* = 0.013 for 40 kHz). These results suggest that the ABR thresholds reached a ceiling after noise exposure.

#### Chronic changes in hearing thresholds at 7 d post-noise exposure

**Figure 6:**
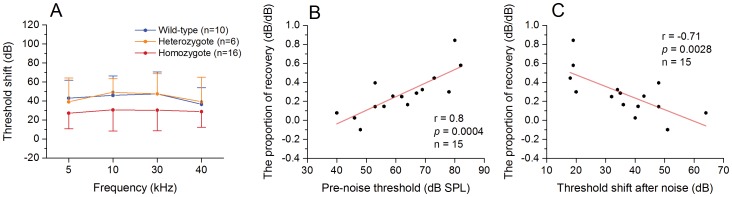
The recovery of ABR thresholds is associated with the level of pre-noise ABR thresholds in the Prestin-CreER^T2^ homozygous mice. **A** Comparison of ABR threshold shifts among the homozygous, heterozygous and WT mice examined at 7 d post-noise exposure. The homozygotes displays less threshold shifts as compared with the heterozygous and WT mice (two-way ANOVA, *F_2,116_* = 16.77, *P*<0.001). **B** Correction of the recovery proportion of ABR thresholds and the pre-noise ABR thresholds. The proportion of ABR recovery is increased with the increase in the level of pre-noise ABR thresholds (Pearson's correction analysis, *r* = 0.8; *p* = 0.0004). The results suggest that the functional status of OHCs before exposure to the noise affects the functional recovery of the cochleae after exposure to the noise. **C** Correction of the recovery of ABR thresholds and the acute threshold shifts examined at 2 h post the acoustic overstimulation. Notice that with the increase in the magnitude of threshold shifts, the proportion of ABR recovery is decreased (Pearson's correction analysis, *r* = −0.71; *p* = 0.0028).

The hearing thresholds partially recovered at 7 d post-noise exposure for the Prestin-CreER^T2^ knockin homozygous mice. The remaining threshold shifts were less in the homozygotes than in the WT and heterozygotes (two-way ANOVA, *F_2,116_* = 16.77, *P*<0.001, Fig. 6A). The average hearing recovery was 8.0±5.7 dB. To determine whether the level of hearing recovery was correlated with the level of pre-noise cochlear dysfunction, we calculated the proportion of hearing recovery per decibel of the acute threshold shift and then examined the Pearson's correlation between the proportions of the recovery and the level of the pre-noise thresholds. We found that the proportion of the recovery was positively correlated with the pre-noise thresholds (Fig. 6B, *r* = 0.8, *p* = 0.0004), suggesting that the ears with cochlear dysfunction had a greater recovery after exposure to the intense noise. We also calculated the correlation between the recovery level and the level of the acute threshold shift and found a negative correlation between the two variables (Fig. 6C, r = −0.71, *p* = 0.0028), suggesting that an improved recovery of cochlear function was related to a reduction in the acute cochlear damage.

### The reduction in threshold shifts of Prestin-CreER^T2^ knockin mice is accompanied by a reduction in the molecular responses of the organ of Corti to acoustic overstimulation

**Figure 7:**
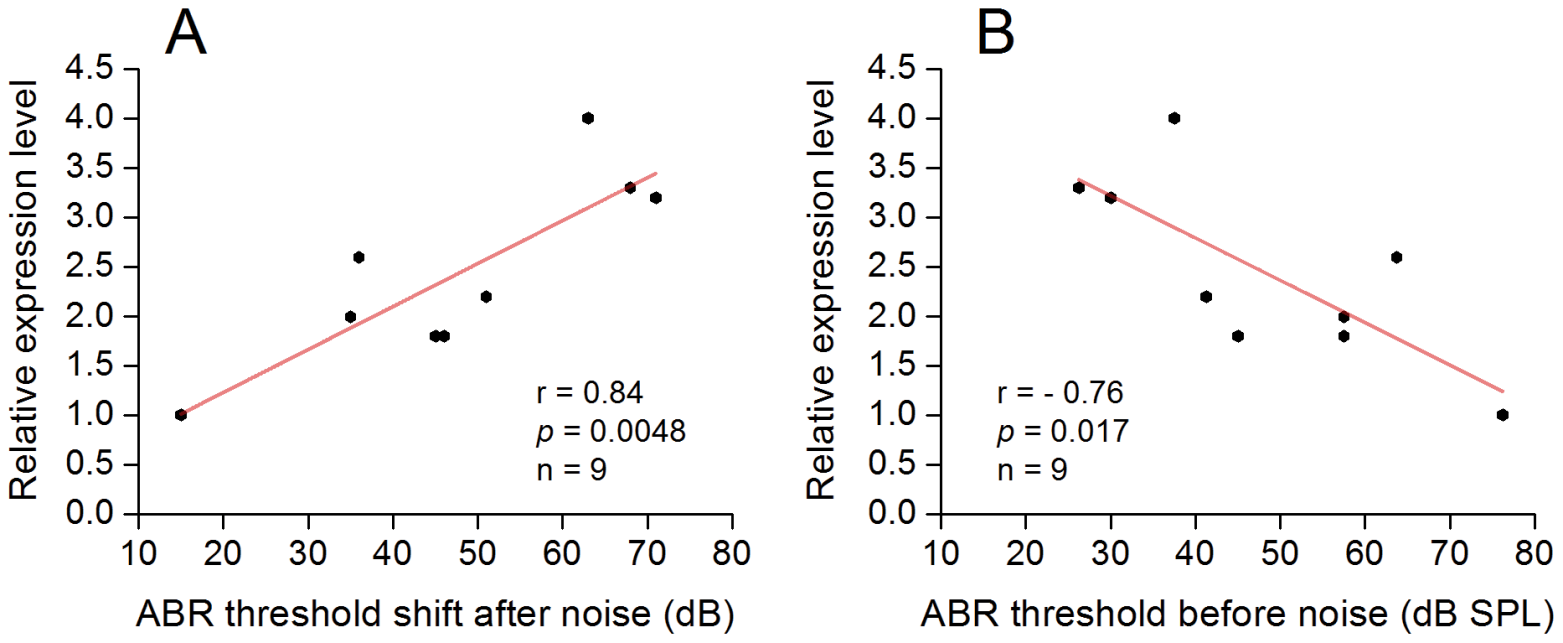
The Prestin-CreER^T2^ knockin homozygous mice display a strong correlation between the levels of *Cdh1* expression and the levels of noise-induced threshold shifts. *Cdh1* expression in the organ of Corti tissues was examined using qRT-PCR. The expression levels are normalized to three reference genes (*Actin*, *GAPDH* and *Hprt1*) and are presented as the fold difference between the prestin expression level and the reference gene expression level. **A** Pearson's correlation analysis reveals a significant positive correlation between the expression level of *Cdh1* and the threshold shifts examined at 2 h after the acoustic injury (Pearson correlation analysis, *r* = 0.84, *p* = 0.0048). **B** Pearson's correlation analysis shows a significant negative correlation between the expression levels of *Cdh1* and the levels of pre-noise ABR thresholds (Fig. 7B; *r* = −0.76, *p* = 0.0172). This result suggests that a greater auditory dysfunction caused by Prestin-CreER^T2^ knockin leads to a lower *cdh1* response to acoustic overstimulation. n =  the number of ears used for the analysis.

The finding of the reduction in noise-induced cochlear dysfunction in homozygous mice prompted us to investigate whether the hearing protection is accompanied by a reduction in the molecular responses of the cochlea to acoustic trauma. *Cdh1* is a member of the classical cadherin family that is expressed in the cell-cell junction of the organ of Corti [24], [29]. Acoustic trauma increases the expression level of the gene at both the transcriptional and protein levels [24]. We therefore used this gene as an indicator of the molecular responses of the cochlea to acoustic injury. We collected the sensory cell-enriched organ of Corti tissue from the homozygous mice at 2 h after the noise exposure and examined the mRNA abundance using qRT-PCR. The *Cdh1* expression level was normalized to the average expression level of three reference genes, *Actin*, *GAPDH* and *Hprt1*. The expression levels of *Cdh1* varied across individual ears. The Pearson's correlation analysis revealed a positive correlation between the levels of *Cdh1* expression and the level of the acute threshold shifts of ABRs (*r* = 0.84, *p* = 0.0048; Fig. 7A), suggesting that the reduction in the threshold shift was accompanied by decreased *Cdh1* responses to the acoustic overstimulation. Moreover, the expression level was negatively correlated with the pre-noise thresholds of the ABRs (Fig. 7B; *r* = −0.76, *p* = 0.0172). Because the up-regulation of *Cdh1* is a molecular response to acoustic overstimulation [24], our results suggest that the Prestin-CreER^T2^ knockin-induced OHC dysfunction reduced the molecular responses of the organ of Corti to acoustic trauma.

### Cochlear pathology

To determine whether OHC dysfunction affects hair cell survival after acoustic trauma, we quantified the numbers of missing sensory cells in Prestin-CreER^T2^ knockin homozygous mice before and two weeks after the noise exposure. We noticed that some samples showed technical defects in the sensory epithelia with the distance >70% from the apex of the cochlea. We therefore excluded the data from this region from the final analysis.

In the cochleae without acoustic trauma, sporadic missing hair cell areas were identified in the organs of Corti (data not shown). After the noise exposure, the number of missing cells was increased. The magnitude of hair cell lesions varied among the individual cochleae, with the missing cell numbers ranging from 8 to 93 per cochlea for the homozygous mice (47±27; mean ± SD; n = 11 cochleae) and 7 to 107 for the heterozygous mice (37±38; n = 6 cochleae). There is no significant difference in the number of missing cells between the homozygous and the heterozygous mice (Mann-Whitney Rank Sun Test, P = 0.025). We further calculated the Pearson's correlation between the level of the pre-noise hearing thresholds and the magnitude of sensory cell damage. There was no correlation between the two variables (r = 0.0003), suggesting that Prestin-CreER^T2^ knockin-induced OHC dysfunction did not affect the process of noise-induced sensory cell death and that the reduction in noise-induced threshold shifts observed in homozygous mice was not mediated by any reduction in sensory cell death.

## Discussion

The current investigation was designed to examine the effect of prestin interference on cochlear responses to acoustic overstimulation. Our data demonstrate that the Prestin-CreER^T2^ knockin results in a threshold elevation of the ABRs with a large individual variation. This reduction in hearing sensitivity is accompanied by a reduction in OHC function, as evidenced by the elevation in DPOAE thresholds and the shift of the input/output function of DPOAE. Our results further demonstrate that the loss of cochlear function reduces the functional impact of acoustic overstimulation. This effect is associated with the level of pre-noise cochlear dysfunction induced by prestin interference. Moreover, our study demonstrates that prestin interference reduces the molecular response of the cochlea to acoustic overstimulation. Together, these observations suggest that the prestin interference leads to the changes in the cochlear stress response to acoustic trauma.

Our mouse model of prestin interference was generated by the insertion of an internal ribosome entry site (IRES)-CreER^T2^–FRT-Neo-FRT cassette into the prestin locus after the stop codon [20]. The initial aim in generating this mouse model was to establish a Cre-model for conditional knockout of OHC genes of interest using a Cre-loxP system. In the previous report [20], both Prestin-CreER^T2^ +/- and Prestin-CreER^T2^ -/- displayed normal hearing sensitivity at 4–6 weeks. However, when the FRT-Neo-FRT cassette was removed by crossing Prestin-CreER^T2^ mice with ACTG-Flpe deletion mice, the animals displayed an average of 25–30 dB hearing loss between 16–32 kHz. The strain that we bred contained the neo components but had hearing loss. This hearing loss is likely associated with OHC dysfunction because the Prestin-CreER^T2^ insertion is prestin-specific and because prestin is expressed exclusively in OHCs [16], This assumption is further supported by the finding of the reduction in DPOAE responses, as well as the comparable level of hearing loss between the Prestin-CreER^T2^ knockin- and prestin-knockout-induced cochlear dysfunction [17], [18].

The question regarding how the Prestin-CreER^T2^-knockin affects the cochlear function is beyond the scope of the current investigation. Clinical observations have documented the phenomenon of asymmetric hearing loss in patients with genetic hearing loss. For example, in a study on patients who had either homozygous or compound heterozygous Cx26 mutations, Cohn and colleagues reported that approximately half of the patients showed asymmetric hearing between ears [30]. In another study on sensorineural hearing loss in children, Smith and colleagues reported that a large portion of the patients displayed asymmetric hearing loss [31]. So far, the molecular mechanisms responsible for such intra-subject variation is not clear. In the current investigation, our expression analyses using qRT-PCR revealed that the prestin expression level was detectable and the expression level was not correlated with the level of hearing sensitivity, suggesting that the hearing dysfunction is not caused by lack of prestin expression. Similar threshold elevations were observed in another independent hypomorphic prestin allele [20]. It remains to be determined whether the Prestin-CreER line used here is a true hypomorphic allele of prestin. No matter what mechanisms are involved, the current investigation demonstrated functional interference due to Cre-knockin. Interestingly, not all animals/cochleae with CreER^T2^ knockin exhibit hearing loss. This finding is consistent with clinical observations of certain genetic hearing loss [32], [33]. Patients who come from the same pedigree and carry the same-disease-causing mutation can display diverse auditory symptoms, possibly due to the influence of epigenetic regulation, environmental factors and genetic modifiers.

The interaction of acoustic trauma with pre-existing cochlear pathologies has been noticed in previous investigations [4], [10], [11]. For subjects with age-related hearing loss, acoustic overstimulation can further compromise the hearing sensitivity. The final outcome of the hearing damage is the sum of the presbycusis and noise-induced hearing loss [34]. For subjects with the pre-existing hearing loss due to acoustic trauma, the impact of subsequent noise exposure is dependent on whether or not the two noise exposures target the same frequency region of the cochlea [1]. When the region of pre-existing damage coincides with the region of second damage, the resulting hearing loss is roughly the loss caused by the second noise alone. When the region of pre-existing damage differs from the region of the second damage, the total damage is the sum of the two individual damages. In our case, the total hearing disability caused by the prestin interference and noise trauma is smaller than that caused by noise alone, suggesting that the pre-existing cochlear dysfunction reduces the subsequent acoustic trauma.

The pre-existing cochlear dysfunction can affect the cochlear responses to acoustic injury in several ways. Many cochlear disease conditions are associated with loss of sensory cells in the cochlea. This structural defect can alter the mechanical properties of the basilar membrane, which in turn affect the response patterns of the basilar membrane to acoustic stimuli. Biological changes, such as the overproduction of reactive oxygen species and the disruption of cellular metabolic activity, can predispose cochlear tissues to acoustic overstimulation [35]. Our current investigation provides evidence that interference of the prestin, which is likely to affect OHC function, reduced the cochlear stress responses to acoustic trauma. OHCs are responsible for the cochlear amplification function [36], [37]. It is likely that the impact of prestin interference is due to the reduction in the mechanical responses of the basilar membrane to acoustic stimuli, which are modulated by OHCs [17], [18], [37].

Two approaches, the modulation of the efferent feedback system and the salicylate treatment, have been used in previous studies to investigate the potential roles of OHCs in modulating the cochlear responses to acoustic trauma. Activation of the efferent system in the cochlea suppresses the OHC feedback force and, thus, the gain of the cochlear amplifier. Electrical stimulation of the efferent pathways in animal studies revealed that a reduction of temporary hearing loss was induced by acoustic overstimulation [38], [39]. Disrupting the efferent function, on the other hand, potentiates noise-induced cochlear damage [40]–[42]. Salicylate, an ototoxic agent that suppresses prestin-based OHC somatic motility [43], [44], causes a dose-dependent temporary threshold shift [45], [46]. The reported effects of salicylate treatment on acoustic overstimulation are inconsistent. Adelman and co-workers reported a reduction in the permanent hearing loss in mice treated with salicylic acid before noise exposure [47]. In contrast, several studies have revealed no detectable effects of salicylate treatment on noise-induced structural and functional changes [48]–[50]. Our current results are consistent with findings that modulating the OHC function alters cochlear stress responses to acoustic overstimulation.

## References

[1] Humes LE (1984) Noise-induced hearing loss as influenced by other agents and by some physical characteristics of the individual. The Journal of the Acoustical Society of America 76:1318–1329. 6512095 10.1121/1.391447

[2] Chen GD (2002) Effect of hypoxia on noise-induced auditory impairment. Hear Res 172:186–195. 12361881 10.1016/s0378-5955(02)00582-8

[3] Fechter LD, Chen GD, Rao D (2002) Chemical Asphyxiants and Noise. Noise Health 4:49–61. 12678928

[4] Li H, Steyger PS (2009) Synergistic ototoxicity due to noise exposure and aminoglycoside antibiotics. Noise Health 11:26–32. 19265251 10.4103/1463-1741.45310 PMC2713742

[5] Fernandez EA, Ohlemiller KK, Gagnon PM, Clark WW (2010) Protection against noise-induced hearing loss in young CBA/J mice by low-dose kanamycin. J Assoc Res Otolaryngol 11:235–244. 20094753 10.1007/s10162-009-0204-9 PMC2862919

[6] Ohlemiller KK, Rybak Rice ME, Rosen AD, Montgomery SC, Gagnon PM (2011) Protection by low-dose kanamycin against noise-induced hearing loss in mice: dependence on dosing regimen and genetic background. Hear Res 280:141–147. 21645602 10.1016/j.heares.2011.05.007 PMC3175505

[7] Miller JM, Dolan DF, Raphael Y, Altschuler RA (1998) Interactive effects of aging with noise induced hearing loss. Scand Audiol Suppl 48: 53–61. 9505298

[8] Shone G, Altschuler RA, Miller JM, Nuttall AL (1991) The effect of noise exposure on the aging ear. Hear Res 56:173–178. 1769911 10.1016/0378-5955(91)90167-8

[9] Sun JC, Bohne BA, Harding GW (1994) Is the older ear more susceptible to noise damage? Laryngoscope 104:1251–1258. 7934596 10.1288/00005537-199410000-00012

[10] Campo P, Subramaniam M, Henderson D (1991) The effect of 'conditioning' exposures on hearing loss from traumatic exposure. Hear Res 55:195–200. 1757287 10.1016/0378-5955(91)90104-h

[11] Subramaniam M, Henderson D, Spongr VP (1993) Protection from noise induced hearing loss: is prolonged 'conditioning' necessary? Hear Res 65:234–239. 8458754 10.1016/0378-5955(93)90216-n

[12] Voldřich L (1979) Noise-noise effect upon the spreading of the posttraumatic progressive necrosis in the organ of Corti. Archives of oto-rhino-laryngology 222:169–173. 444150 10.1007/BF00456312

[13] McFadden SL, Ohlemiller KK, Ding D, Shero M, Salvi RJ (2001) The Influence of Superoxide Dismutase and Glutathione Peroxidase Deficiencies on Noise-Induced Hearing Loss in Mice. Noise Health 3:49–64. 12689448

[14] Hakuba N, Koga K, Gyo K, Usami SI, Tanaka K (2000) Exacerbation of noise-induced hearing loss in mice lacking the glutamate transporter GLAST. J Neurosci 20:8750–8753. 11102482 10.1523/JNEUROSCI.20-23-08750.2000 PMC6773045

[15] Kozel PJ, Davis RR, Krieg EF, Shull GE, Erway LC (2002) Deficiency in plasma membrane calcium ATPase isoform 2 increases susceptibility to noise-induced hearing loss in mice. Hear Res 164:231–239. 11950541 10.1016/s0378-5955(01)00420-8

[16] Zheng J, Shen W, He DZ, Long KB, Madison LD, et al (2000) Prestin is the motor protein of cochlear outer hair cells. Nature 405:149–155. 10821263 10.1038/35012009

[17] Liberman MC, Gao J, He DZ, Wu X, Jia S, et al (2002) Prestin is required for electromotility of the outer hair cell and for the cochlear amplifier. Nature 419:300–304. 12239568 10.1038/nature01059

[18] Wu X, Gao J, Guo Y, Zuo J (2004) Hearing threshold elevation precedes hair-cell loss in prestin knockout mice. Brain Res Mol Brain Res 126:30–37. 15207913 10.1016/j.molbrainres.2004.03.020

[19] He DZ, Jia S, Sato T, Zuo J, Andrade LR, et al (2010) Changes in plasma membrane structure and electromotile properties in prestin deficient outer hair cells. Cytoskeleton 67:43–55. 20169529 10.1002/cm.20423 PMC2842980

[20] Fang J, Zhang WC, Yamashita T, Gao J, Zhu MS, et al (2012) Outer hair cell-specific prestin-CreERT2 knockin mouse lines. Genesis 50:124–131. 21954035 10.1002/dvg.20810 PMC3261330

[21] Hu BH, Cai Q, Hu Z, Patel M, Bard J, et al (2012) Metalloproteinases and their associated genes contribute to the functional integrity and noise-induced damage in the cochlear sensory epithelium. J Neurosci 32:14927–14941. 23100416 10.1523/JNEUROSCI.1588-12.2012 PMC3521496

[22] Cai Q, Whitcomb C, Eggleston J, Sun W, Salvi R, et al**.** (2013) Round window closure affects cochlear responses to suprathreshold stimuli. Laryngoscope 123. 10.1002/lary.24394 PMC4219855 24114866

[23] Hu BH, Cai Q (2010) Acoustic overstimulation modifies Mcl-1 expression in cochlear sensory epithelial cells. J Neurosci Res 88:1812–1821. 20091770 10.1002/jnr.22333 PMC3521497

[24] Cai Q, Patel M, Coling D, Hu BH (2012) Transcriptional changes in adhesion-related genes are site-specific during noise-induced cochlear pathogenesis. Neurobiology of disease 45:723–732. 22044737 10.1016/j.nbd.2011.10.018 PMC3259216

[25] Cai Q, Wang B, Patel M, Yang SM, Hu BH (2013) RNAlater facilitates microdissection of sensory cell-enriched samples from the mouse cochlea for transcriptional analyses. J Neurosci Methods 219:240–251. 23958750 10.1016/j.jneumeth.2013.08.010 PMC3836215

[26] Hu BH, Henderson D, Nicotera TM (2002) F-actin cleavage in apoptotic outer hair cells in chinchilla cochleas exposed to intense noise. Hear Res 172:1–9. 12361861 10.1016/s0378-5955(01)00361-6

[27] Cheatham MA, Huynh KH, Gao J, Zuo J, Dallos P (2004) Cochlear function in Prestin knockout mice. J Physiol 560:821–830. 15319415 10.1113/jphysiol.2004.069559 PMC1665294

[28] Yamashita T, Fang J, Gao J, Yu Y, Lagarde MM, et al (2012) Normal hearing sensitivity at low-to-middle frequencies with 34% prestin-charge density. PloS one 7:e45453. 23029017 10.1371/journal.pone.0045453 PMC3448665

[29] Whitlon DS (1993) E-cadherin in the mature and developing organ of Corti of the mouse. J Neurocytol 22:1030–1038. 8106878 10.1007/BF01235747

[30] Cohn ES, Kelley PM, Fowler TW, Gorga MP, Lefkowitz DM, et al (1999) Clinical studies of families with hearing loss attributable to mutations in the connexin 26 gene (GJB2/DFNB1). Pediatrics 103:546–550. 10049954 10.1542/peds.103.3.546

[31] Smith RJ, Bale JF Jr, White KR (2005) Sensorineural hearing loss in children. Lancet 365:879–890. 15752533 10.1016/S0140-6736(05)71047-3

[32] Zhu Y, Huang S, Kang D, Han M, Wang G, et al (2014) Analysis of the heteroplasmy level and transmitted features in hearing-loss pedigrees with mitochondrial 12S rRNA A1555G mutation. BMC genetics 15:26. 24533451 10.1186/1471-2156-15-26 PMC3933286

[33] Cheng J, Zhu Y, He S, Lu Y, Chen J, et al (2011) Functional mutation of SMAC/DIABLO, encoding a mitochondrial proapoptotic protein, causes human progressive hearing loss DFNA64. Am J Hum Genet 89:56–66. 21722859 10.1016/j.ajhg.2011.05.027 PMC3135809

[34] Lebo CP, Reddell RC (1972) The presbycusis component in occupational hearing loss. The Laryngoscope 82:1399–1409. 4262332 10.1288/00005537-197208000-00002

[35] Henderson D, Bielefeld EC, Harris KC, Hu BH (2006) The Role of Oxidative Stress in Noise-Induced Hearing Loss. Ear Hear 27:1–19. 16446561 10.1097/01.aud.0000191942.36672.f3

[36] Brownell WE, Bader CR, Bertrand D, de Ribaupierre Y (1985) Evoked mechanical responses of isolated cochlear outer hair cells. Science 227:194–196. 3966153 10.1126/science.3966153

[37] Dallos P (2008) Cochlear amplification, outer hair cells and prestin. Current opinion in neurobiology 18:370–376. 18809494 10.1016/j.conb.2008.08.016 PMC2630119

[38] Rajan R (1988) Effect of electrical stimulation of the crossed olivocochlear bundle on temporary threshold shifts in auditory sensitivity. I. Dependence on electrical stimulation parameters. J Neurophysiol 60:549–568. 3171641 10.1152/jn.1988.60.2.549

[39] Reiter ER, Liberman MC (1995) Efferent-mediated protection from acoustic overexposure: relation to slow effects of olivocochlear stimulation. J Neurophysiol 73:506–514. 7760114 10.1152/jn.1995.73.2.506

[40] Puel JL, Bobbin RP, Fallon M (1988) An ipsilateral cochlear efferent loop protects the cochlea during intense sound exposure. Hear Res 37:65–69. 3225232 10.1016/0378-5955(88)90078-0

[41] Zheng XY, Henderson D, McFadden SL, Hu BH (1997) The role of the cochlear efferent system in acquired resistance to noise-induced hearing loss. Hear Res 104:191–203. 9119763 10.1016/s0378-5955(96)00187-6

[42] Kujawa SG, Liberman MC (1997) Conditioning-related protection from acoustic injury: effects of chronic deefferentation and sham surgery. J Neurophysiol 78:3095–3106. 9405529 10.1152/jn.1997.78.6.3095

[43] Dieler R, Shehata-Dieler WE, Brownell WE (1991) Concomitant salicylate-induced alterations of outer hair cell subsurface cisternae and electromotility. J Neurocytol 20:637–653. 1940979 10.1007/BF01187066

[44] Shehata WE, Brownell WE, Dieler R (1991) Effects of salicylate on shape, electromotility and membrane characteristics of isolated outer hair cells from guinea pig cochlea. Acta Otolaryngol 111:707–718. 1950533 10.3109/00016489109138403

[45] Oliver D, He DZ, Klocker N, Ludwig J, Schulte U, et al (2001) Intracellular anions as the voltage sensor of prestin, the outer hair cell motor protein. Science 292:2340–2343. 11423665 10.1126/science.1060939

[46] Santos-Sacchi J, Song L, Zheng J, Nuttall AL (2006) Control of mammalian cochlear amplification by chloride anions. J Neurosci 26:3992–3998. 16611815 10.1523/JNEUROSCI.4548-05.2006 PMC6673883

[47] Adelman C, Freeman S, Paz Z, Sohmer H (2008) Salicylic acid injection before noise exposure reduces permanent threshold shift. Audiol Neurootol 13:266–272. 18259079 10.1159/000115436

[48] Woodford CM, Henderson D, Hamernik RP (1978) Effects of combinations of sodium salicylate and noise on the auditory threshold. Ann Otol Rhinol Laryngol 87:117–127. 623409 10.1177/000348947808700119

[49] Bancroft BR, Boettcher FA, Salvi RJ, Wu J (1991) Effects of noise and salicylate on auditory evoked-response thresholds in the chinchilla. Hearing research 54:20–28. 1917714 10.1016/0378-5955(91)90132-s

[50] Spongr VP, Boettcher FA, Saunders SS, Salvi RJ (1992) Effects of noise and salicylate on hair cell loss in the chinchilla cochlea. Archives of Otolaryngology—Head & Neck Surgery 118:157. 1540346 10.1001/archotol.1992.01880020051015

